# Efficacy and Safety of Switching from Nalfurafine to Difelikefalin in Hemodialysis Patients with Refractory Pruritus

**DOI:** 10.3390/jpm16100489

**Published:** 2026-09-23

**Authors:** Yoshimasa Sakurabu, Haruhito A. Uchida, Kumiko Tokunaga, Yuko Ogiso, Miwako Motoie, Megumi Yoshitake, Taro Sugimoto, Jun Wada

**Affiliations:** 1 Department of Nephrology, Rheumatology, Endocrinology and Metabolism, Okayama University Graduate School of Medicine, Dentistry and Pharmaceutical Sciences, Okayama 700-8558, Japan; p41b7lme@s.okayama-u.ac.jp (Y.S.); junwada@okayama-u.ac.jp (J.W.); 2 Sugimoto Clinic, Okayama 719-1136, Japan; toku0149@gmail.com (K.T.); koigokoro178-54@softbank.ne.jp (Y.O.); sc-touseki8886@kym.biglobe.ne.jp (M.M.); huimeijiwu@gmail.com (M.Y.); staro@mxa.mesh.ne.jp (T.S.)

**Keywords:** hemodialysis-associated pruritus, difelikefalin, kappa-opioid receptor agonist, refractory pruritus, nalfurafine

## Abstract

**Background**: Hemodialysis-associated pruritus is common and can substantially impair quality of life. Given its heterogeneous pathophysiology and variable treatment response, individualized therapeutic strategies are needed. This study investigated the prevalence and severity of pruritus and clinical outcomes after switching from nalfurafine to difelikefalin. **Methods**: Clinical data from 108 maintenance hemodialysis patients whose pruritus severity was assessed using the Numerical Rating Scale (NRS) were retrospectively reviewed. Ten patients with persistent moderate-to-severe pruritus (NRS ≥ 3) despite treatment with nalfurafine were switched to difelikefalin in routine clinical practice, of whom eight completed the 16-week follow-up and were included in the switch group. Eight patients matched for age, sex, dialysis vintage, and baseline NRS served as controls. Changes in NRS scores at weeks 4 and 16, as well as adverse events, were evaluated. **Results**: Among 108 patients, 21 (20%) had no pruritus, while 20 (18.5%), 47 (43.5%), and 20 (18.5%) had mild, moderate, and severe pruritus, respectively. Of 14 patients receiving nalfurafine, 13 (92.9%) had persistent moderate-to-severe pruritus. In the switch group, mean NRS decreased from 5.9 ± 1.7 at baseline to 2.3 ± 2.3 at week 4 and 2.0 ± 0.9 at week 16 (*p* = 0.0156 and *p* = 0.0078, respectively), with no meaningful change in controls. No serious adverse events strongly related to difelikefalin were identified. **Conclusions**: Pruritus was highly prevalent in this cohort. Switching to difelikefalin was associated with rapid and sustained improvement in patients inadequately responsive to nalfurafine, with favorable tolerability, supporting its potential role as an individualized treatment option.

## 1. Introduction

Chronic kidney disease (CKD) poses a major burden on healthcare systems globally, and the progression from CKD to end-stage kidney disease (ESKD) is characterized by histological tubulointerstitial fibrosis, regardless of the underlying cause [1]. Fibrosis alters the normal renal architecture through excessive extracellular matrix accumulation within the renal parenchyma, ultimately requiring kidney replacement therapy when conservative management is no longer feasible [2]. Although the annual number of incident hemodialysis patients has recently trended downward, the total number of patients receiving chronic dialysis in Japan reached approximately 347,000 at the end of 2021, with the vast majority undergoing hemodialysis [3]. Etiologies are diverse, including diabetic nephropathy, and managing complications such as cardiovascular events in an aging population with complex clinical backgrounds has become a critical clinical challenge [4]. Consequently, treating hemodialysis patients with diverse backgrounds requires individualization based on clinical profiles rather than a uniform therapeutic approach.

Hemodialysis patients are prone to complications, such as CKD-mineral and bone disorder (CKD-MBD) and heart failure, which directly affect survival and profoundly impact quality of life (QOL). In recent years, improving patient QOL has been recognized as a vital therapeutic goal alongside extending overall survival. Among these complications, CKD-associated pruritus (CKD-aP) is a representative symptom that markedly impairs patient QOL. Pruritus is present in approximately 60–80% of hemodialysis patients, with 20–35% or more reporting moderate-to-severe itch, which serves as an independent predictor of mortality [5,6]. Because pruritus is a subjective symptom, assessment using the numerical rating scale (NRS) is widely adopted. However, owing to the lack of objective biomarkers and the heterogeneous pathophysiology of the condition, no established gold-standard treatment exists to date [7]. Therefore, detailed clinical profiling of each patient’s underlying disease and subjective burden, followed by tailored therapeutic interventions, is essential.

The etiology of CKD-aP is multifactorial, involving xerosis, accumulation of pruritogenic substances, and an imbalance in the endogenous opioid system. Conventional management has achieved partial success through combination therapies involving moisturizers, oral or topical antihistamines [8], and the introduction of existing kappa-opioid receptor (KOR) agonists such as nalfurafine. Although nalfurafine is an effective oral KOR agonist, its central nervous system (CNS) permeability often makes safe dose escalation difficult in non-responders due to concerns regarding CNS-related adverse effects, such as somnolence. Consequently, a substantial proportion of patients exhibit treatment resistance despite multimodal therapy, representing an unresolved clinical need for new therapeutic strategies. Recently, difelikefalin, a novel intravenous agent that selectively acts on peripheral KORs with minimal blood–brain barrier permeability, has emerged as a new therapeutic option. Although this novel agent features a mechanism expected to reduce CNS side effects, real-world data regarding the clinical efficacy and adverse event profiles associated with switching from oral nalfurafine to intravenous difelikefalin remain limited. In particular, reports evaluating the efficacy and safety of switching to difelikefalin in patients with persistent pruritus during nalfurafine therapy are scarce [9]. While Takahashi et al. [9] conducted a prospective interventional study, the present retrospective analysis provides complementary real-world evidence from routine clinical practice and extends follow-up to 16 weeks.

Therefore, this study aimed to determine the prevalence and severity of pruritus among maintenance hemodialysis patients at our clinic and to clarify the clinical status of refractory cases with persistent symptoms despite receiving existing therapies, particularly nalfurafine. Furthermore, we aimed to investigate the efficacy and safety of switching to difelikefalin in these refractory cases to provide insights that could contribute to the establishment of novel therapeutic strategies. Given the individual variations in pathophysiology, drug responsiveness, and side-effect susceptibility in CKD-aP, an approach that switches patients to optimal agents considering treatment response and risk profiles represents an important real-world application of personalized medicine in dialysis.

## 2. Materials and Methods

### 2.1. Study Design

This single-center retrospective observational study comprised two predefined components: a cross-sectional assessment of pruritus prevalence and severity among all eligible maintenance hemodialysis patients and a retrospective longitudinal evaluation of the efficacy and safety of switching from nalfurafine to difelikefalin in patients with refractory pruritus. This study included all 108 patients receiving maintenance hemodialysis as outpatients at our clinic between January and May 2025. All patients undergoing maintenance hemodialysis at our clinic during the study period were investigated for the cross-sectional assessment. Demographic and clinical information was retrospectively extracted from medical records, including age, sex, dialysis vintage, concomitant medications, laboratory values, nalfurafine use, difelikefalin dose and treatment duration, adverse events, and treatment discontinuation status with reasons. Pruritus intensity was extracted and evaluated using questionnaires or symptom assessment forms recorded and accumulated as part of routine clinical care. Evaluation time points were defined as before treatment change, 4 weeks after treatment change, and 16 weeks after treatment change, and available data were analyzed in accordance with real-world clinical documentation. To determine the prevalence and intensity of pruritus, a descriptive questionnaire was administered to all eligible outpatients during the predefined study period. For patients who had difficulty completing the form independently, attending nurses conducted oral interviews and transcribed the responses. Itch intensity was assessed using the 11-point NRS (0–10). Based on established consensus cutoff values, pruritus severity was categorized as mild (NRS < 3), moderate (3 ≤ NRS < 7), or severe (NRS ≥ 7) in accordance with the previous report [10]. Among all patients, fourteen patients were identified those currently receiving nalfurafine for pruritus. Of these 14 patients, 1 (7.1%) had mild pruritus (NRS 1–2), 7 (50.0%) had moderate pruritus (NRS 3–6), and 6 (42.9%) had severe pruritus (NRS ≥ 7).

For the retrospective longitudinal component, ten patients were confirmed who had persistent moderate-to-severe pruritus (NRS ≥ 3) despite nalfurafine therapy and were switched from nalfurafine to difelikefalin because they reported that pruritus interfered with their daily activities during clinical consultation. In accordance with the Japanese package insert guidelines, difelikefalin was administered intravenously via the venous line of the dialysis circuit at the end of each hemodialysis session (three times weekly) during blood reinfusion. The dose was determined based on dry weight (DW): 17.5 µg for DW < 45 kg, 25.0 µg for DW 45 to <65 kg, 35.0 µg for DW 65 to <85 kg, and 42.5 µg for DW ≥85 kg. If a patient’s DW changed during the observation period, the difelikefalin dose was adjusted accordingly based on the updated DW. The efficacy and safety of switching to difelikefalin were evaluated in these patients. Safety was assessed according to the severity of adverse events after the medication change. NRS evaluations were performed at baseline (week 0), week 4, and week 16 after switching. Of the 14 patients receiving nalfurafine, four were not switched to difelikefalin during the observation period; one patient had mild pruritus, indicating adequate symptom control with nalfurafine, whereas the remaining three patients had moderate-to-severe pruritus based on NRS assessment but reported no impairment in daily activities; therefore, after consultation with their attending physicians, their treatment was not changed. Of the 10 patients who initiated difelikefalin switch therapy, two were excluded from the efficacy analysis because of hospitalization before the 4-week evaluation (*n* = 1) or treatment discontinuation due to intradialytic hypotension before NRS evaluation (*n* = 1). These exclusion reasons were recorded before the final efficacy dataset was defined. Ultimately, eight patients who switched to difelikefalin and completed efficacy and safety evaluations at 4 and 16 weeks were assigned as the switch group. In addition, among all 108 patients, 73 had pruritus but were not receiving nalfurafine. To minimize the potential influence of seasonal variation in pruritus severity over time, patients with an NRS score ≥ 3 who were receiving neither nalfurafine nor difelikefalin were considered as candidates for the control group. From this cohort, a control group of eight patients was selected by matching for age (±5 years), sex, dialysis vintage (±2 years), and baseline NRS score (±1 point) to reduce confounding by major baseline characteristics and to account for potential seasonal variation over the same observation period [Figure 1].

This study was approved by the Ethics Committee of Sugimoto Clinic (approval no. SC-2025-0228, approved 2 October 2025) and was conducted in accordance with institutional guidelines and the principles of the Declaration of Helsinki.

### 2.2. Endpoints

The primary endpoint was the change in the NRS pruritus score (0–10) from baseline (prior to difelikefalin switch) to 4 and 16 weeks post-switch. The NRS was self-reported by patients, where 0 represented “no itch” and 10 represented “worst imaginable itch.”

The secondary endpoints included the distribution of pruritus severity categories at each evaluation time point. Severity was defined as mild (NRS < 3), moderate (3 ≤ NRS < 7), or severe (NRS ≥ 7).

Safety endpoints included the incidence and severity of adverse events during the treatment period. The adverse events of interest were dizziness, somnolence, headache, hot flashes, constipation, diarrhea, fatigue, altered mental status, and nausea. Severity was graded as 0 (none), 1 (mild), 2 (moderate), or 3 (severe). For events leading to treatment discontinuation, the timing of onset, clinical course, and causal relationship with difelikefalin were reviewed using electronic medical records.

### 2.3. Statistical Analysis

Statistical analyses were performed using JMP Student Edition 19 version19.1.3 (JMP Statistical Discovery LLC, Cary, NC, USA). Continuous variables and NRS scores are presented as mean ± standard deviation. Given the small sample size (*n* = 8 per group) and the ordinal nature of NRS scores, non-parametric methods were utilized for all efficacy evaluations. Within-group longitudinal changes across the three evaluation time points (baseline, week 4, and week 16) were assessed using the Friedman test, followed by Wilcoxon signed-rank tests for post hoc pairwise comparisons relative to baseline. Direct between-group comparisons between the switch and control groups at each timepoint, as well as for NRS score reductions from baseline, were performed using Mann–Whitney U tests. A two-sided *p*-value < 0.05 was considered statistically significant.

## 3. Results

### 3.1. Cross-Sectional Survey

Among the 108 hemodialysis patients, 21 (20%) had no pruritus, 20 (18%) had mild pruritus (NRS 1–2), 47 (43.5%) had moderate pruritus (NRS 3–6), and 20 (19%) had severe pruritus (NRS ≥ 7) [Figure 2]. Overall, 72 patients (66.7%) were receiving therapeutic interventions for pruritus, whereas 36 patients (33.3%) were untreated. Specifically, 49 patients (45.4%) were treated with topical agents, including heparinoid moisturizers or crotamiton/hydrocortisone combination creams, and 34 (31.5%) received oral antihistamines, with 13 patients receiving combination therapy with both topical agents and oral antihistamines (2 agents). Additionally, 2 patients received nalfurafine monotherapy without concomitant topical or oral agents. Furthermore, 35 (32.4%) underwent online hemodiafiltration as their primary dialysis modality.

Fourteen of the 108 patients were currently taking oral nalfurafine. Among these 14 patients, 1 (7.1%) reported mild, 7 (50.0%) reported moderate, and 6 (42.9%) reported severe pruritus. Thus, 13 patients (92.9%) receiving nalfurafine suffered from refractory pruritus with persistent moderate-to-severe symptoms. Among those on nalfurafine, 10 patients (71.4%) used topical treatments, 10 (71.4%) used oral antihistamines, and 9 (64.3%) underwent online HDF. Five patients (35.7%) received all three concurrent interventions [Figure 3].

In contrast, among the 94 patients not receiving nalfurafine, 21 (22.3%) had no pruritus (NRS 0), whereas pruritus severity was mild in 19 (20.2%), moderate in 40 (42.6%), and severe in 14 (14.9%). Consequently, 54 patients (57.4% of the non-nalfurafine group) had persistent moderate-to-severe pruritus (NRS ≥ 3). Reasons for not taking nalfurafine in this cohort included prior discontinuation due to side effects, a clinical strategy to minimize polypharmacy, or lack of explicit daily activity disruption despite persistent itching [Figure 4].

### 3.2. Baseline Patient Characteristics

The baseline characteristics of the switch (*n* = 8) and control (n = 8) groups are summarized in Table 1. The evaluated variables included age, sex, dry weight, dialysis vintage, serum laboratory parameters (hemoglobin, albumin, blood urea nitrogen, creatinine, corrected calcium, phosphate, and intact parathyroid hormone), and baseline NRS scores. Baseline NRS scores were 5.9 ± 1.7 in the switch group and 4.6 ± 1.3 in the control group, indicating moderate pruritus in both cohorts. In the switch group, mean corrected calcium (9.3 ± 0.5 mg/dL) and phosphate (5.1 ± 0.9 mg/dL) levels were within the target ranges specified by the Japanese CKD-MBD guidelines [11]. Although mineral metabolism abnormalities can contribute to CKD-associated pruritus [12,13], no statistically significant differences were observed between the switch and control groups in laboratory parameters, age, sex, dialysis vintage, or baseline NRS scores [Table 1].

### 3.3. Longitudinal Changes in Pruritus Severity (Figure 5, Figure 6 and Figure 7)

In the switch group, mean ± SD NRS scores decreased from 5.9 ± 1.7 at baseline to 2.3 ± 2.3 at week 4 and 2.0 ± 0.9 at week 16. Non-parametric evaluation confirmed a significant overall longitudinal change over time (Friedman test, *p* = 0.0036). Post hoc pairwise comparisons using the Wilcoxon signed-rank test demonstrated statistically significant reductions in NRS scores at both week 4 (*p* = 0.0156) and week 16 (*p* = 0.0078) compared with baseline. Furthermore, patients who showed limited improvement at week 4 demonstrated further reductions in NRS scores by week 16 with continued therapy.

Regarding individual patient trajectories, one patient whose NRS score improved from severe pruritus (7) to moderate pruritus (3) at week 4 showed a slight increase to 4 at week 16. Two patients who achieved complete resolution (NRS 0) at week 4 showed slight increases to 1 and 2 at week 16, respectively. Nevertheless, at week 16, all patients in the switch group maintained improvements in NRS scores relative to baseline, with all scores ranging from 0 to 5 and no patients having severe pruritus (NRS ≥ 7).

Conversely, in the control group, mean NRS scores progressed from 4.6 ± 1.3 at baseline to 5.6 ± 2.3 at week 4 and 4.5 ± 0.9 at week 16. No statistically significant changes were observed at week 4 (Wilcoxon signed-rank test, *p* = 0.2344) or week 16 (*p* = 0.2813).

Direct between-group comparisons using Mann–Whitney U tests further confirmed the superiority of the difelikefalin switch over continued prior therapy. Baseline NRS scores were comparable between the switch and control groups (*p* = 0.1455). However, absolute NRS scores were significantly lower in the switch group than in the control group at both week 4 (*p* = 0.0221) and week 16 (*p* = 0.0252). Furthermore, score reductions from baseline were markedly greater in the switch group compared with the control group at week 4 (*p* = 0.0036) and week 16 (*p* = 0.0097).

These results indicate that switching to difelikefalin provided significant and sustained itch reduction into the mild range through week 16, whereas no spontaneous resolution occurred in the control cohort.

**Figure 5:**
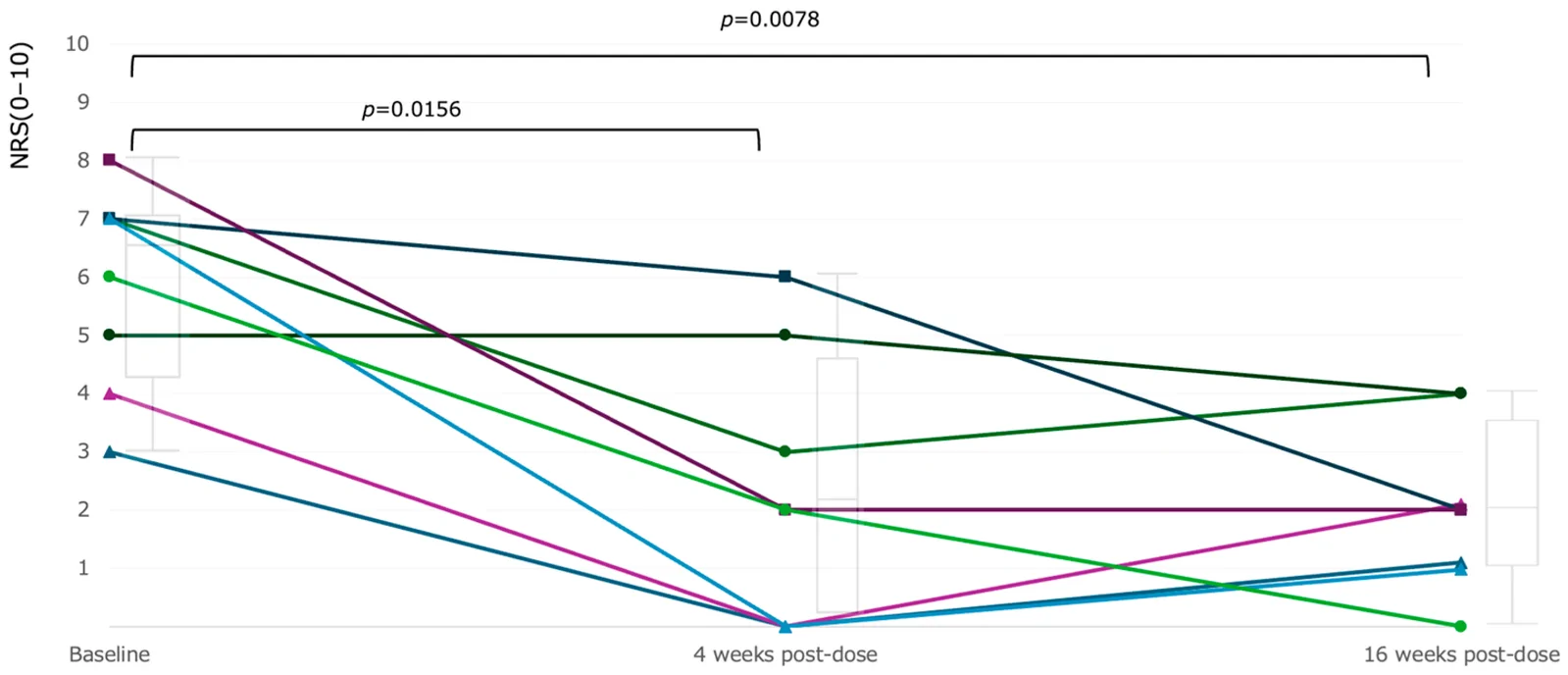
**The group switched to difelikefalin.** Individual NRS scores in the switch group are shown at baseline, week 4, and week 16 after switching from nalfurafine to difelikefalin. Each line represents one patient. Overall longitudinal change across timepoints was evaluated using the Friedman omnibus test (*p* = 0.0036). *p*-values for pairwise comparisons relative to baseline were calculated using post hoc Wilcoxon signed-rank tests (*p* = 0.0156 at week 4; *p* = 0.0078 at week 16). NRS, numerical rating scale.

**Figure 6:**
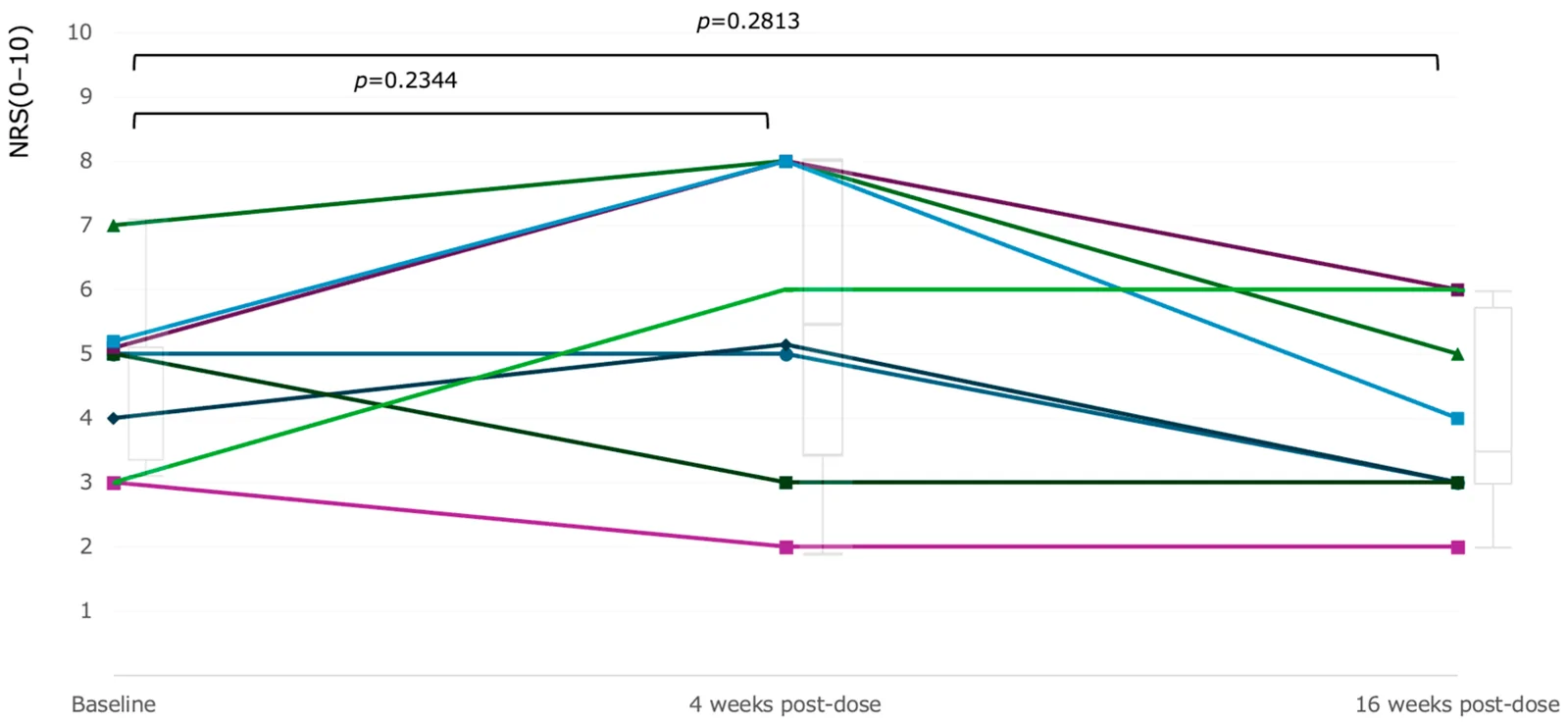
**The control group.** Individual NRS scores in the control group are shown at baseline, week 4, and week 16. Each line represents one patient. Within-group pairwise comparisons relative to baseline were evaluated using Wilcoxon signed-rank tests (*p* = 0.2344 at week 4; *p* = 0.2813 at week 16). NRS, numerical rating scale.

**Figure 7:**
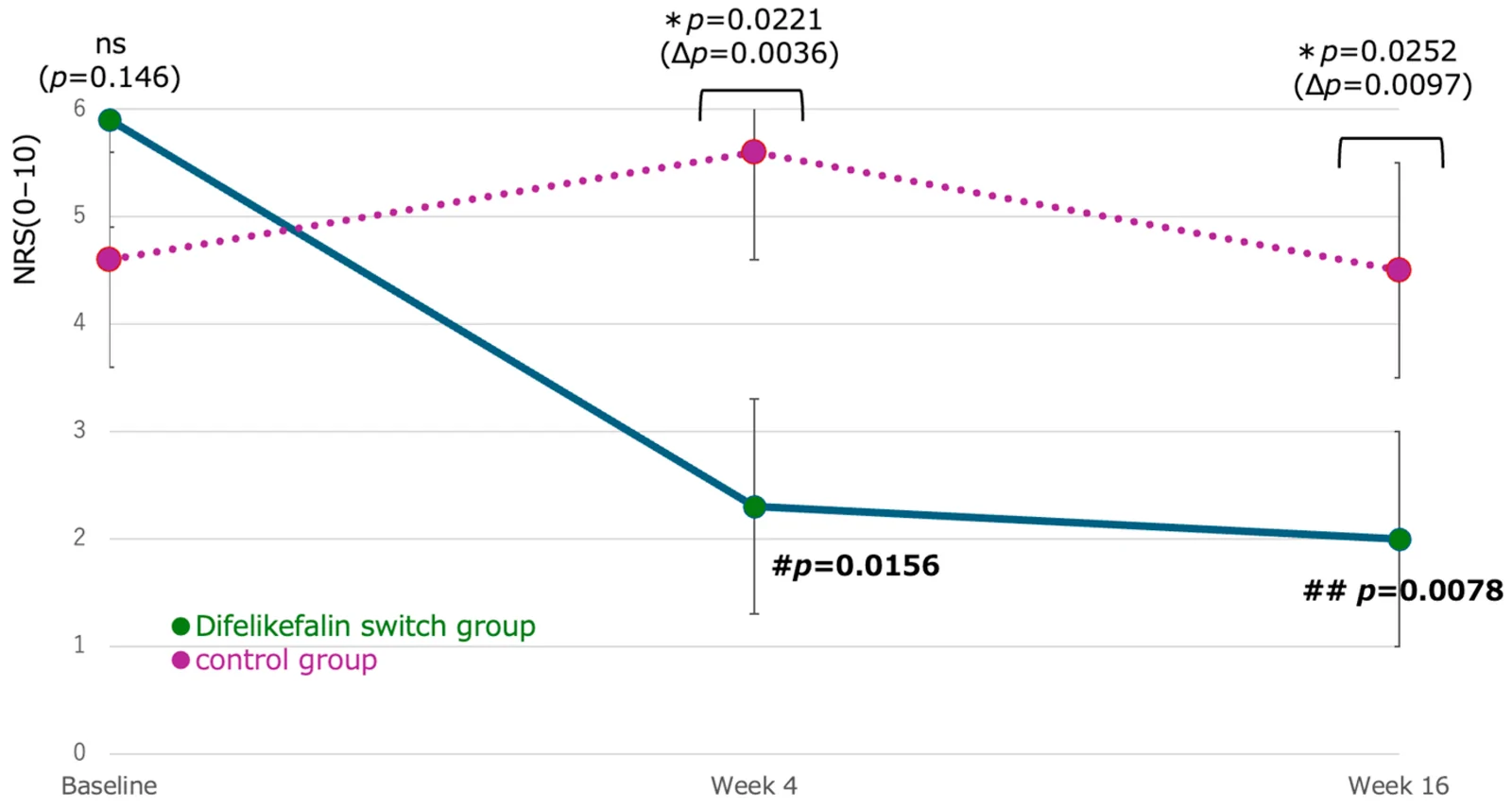
**Changes in Itch Intensity: Switch Group vs. Control Group Over 16 Weeks.** Mean ± SD NRS scores at baseline, week 4, and week 16 in the switch group (*n* = 8) and control group (*n* = 8). Direct between-group comparisons at each timepoint were evaluated using Mann–Whitney U tests (* *p* = 0.0221 at week 4, *p* = 0.0252 at week 16 vs. control group). Within-group longitudinal changes relative to baseline were assessed using Friedman tests followed by Wilcoxon signed-rank tests for post hoc pairwise comparisons. NRS, numerical rating scale. ＊ Between-group comparison by Mann–Whitney U test (absolute NRS score). Δ Between-group comparison of score reduction from baseline(Mann–Whitney U test). # Within-group pairwise comparison vs. baseline by Wilcoxon signed-rank test (# *p* = 0.0156 at week 4, ## *p* = 0.0078 at week 16). Data presented as mean ± SD. ns: not significant.

### 3.4. Safety and Adverse Events (Table 2)

Adverse events recorded 1 month after switching to difelikefalin are shown in Table 2. One patient experienced worsening somnolence from moderate to severe; however, this event coincided with the initiation of pregabalin at another institution for vascular headache. Somnolence resolved rapidly after pregabalin discontinuation, suggesting a stronger causal association with pregabalin than with difelikefalin.

Other reported events included fatigue, mild anxiety/mental status changes, and mild nausea (grade 1), none of which impaired patient QOL or dialysis continuation. Existing baseline symptoms, such as pre-switch fatigue and constipation, showed no clear aggravation, and no novel treatment-emergent AEs directly attributable to medication changes were identified.

One patient discontinued difelikefalin early because of intradialytic hypotension. Notably, this patient had a history of transient intradialytic hypotension prior to initiating difelikefalin, and the frequency of hypotensive episodes increased rather than improved after difelikefalin discontinuation. Subsequent diagnostic workup revealed severe mitral regurgitation, which was successfully treated with transcatheter edge-to-edge repair (MitraClip^®^ (Abbott Vascular, Santa Clara, CA, USA)) at a tertiary cardiology center. Additionally, coronary angiography identified 99% stenosis in the left anterior descending artery, requiring percutaneous coronary intervention (PCI). Following these cardiac interventions, intradialytic hypotension completely resolved. Based on this clinical sequence, a direct causal relationship between difelikefalin and intradialytic hypotension was deemed unlikely.

No adverse events considered attributable to difelikefalin were observed after week 4, and all patients in the switch group were able to continue difelikefalin treatment through week 16. No severe adverse events considered strongly related to difelikefalin or adverse events leading to treatment withdrawal occurred during the observation period.

**Table 2:** Adverse event occurrence and changes 1 month post-switch.

Patient	Dizziness/ Floating Sensation	Somnolence	Headache	Hot Flashes	Constipation	Diarrhea	Fatigue	Altered Mental Status	Nausea
A	0 → 0	0 → 0	0 → 0	0 → 0	0 → 0	0 → 0	0 → 0	0 → 0	0 → 0
B	0 → 0	0 → 3 ＊	0 → 0	0 → 0	0 → 0	0 → 0	0 → 1	0 → 1	0 → 1
C	0 → 0	0 → 0	0 → 0	0 → 0	0 → 0	0 → 0	0 → 0	0 → 0	0 → 0
D	0 → 0	0 → 0	0 → 0	0 → 0	1 → 1	0 → 0	0 → 0	0 → 0	0 → 0
E	1 → 0	0 → 0	0 → 0	0 → 0	0 → 0	0 → 0	1 → 0	0 → 0	0 → 0
F	0 → 0	0 → 0	0 → 0	0 → 0	1 → 1	0 → 0	2 → 2	1 → 1	0 → 0
G	0 → 0	1 → 1	0 → 0	0 → 0	0 → 0	0 → 0	0 → 0	0 → 0	0 → 0
H	0 → 0	2 → 2	0 → 0	0 → 0	2 → 2	0 → 0	1 → 1	0 → 0	0 → 0

Grade criteria: 0 = None, 1 = Mild, 2 = Moderate, 3 = Severe. Transitions in adverse event severity grade are shown as “Baseline (pre-switch) → 1 month post-switch.” ＊ Supplementary information: Regarding the “somnolence (Grade 3)” in Patient B, improvement was observed following the discontinuation of pregabalin.

## 4. Discussion

This study demonstrated that switching from nalfurafine to difelikefalin in hemodialysis patients with refractory pruritus despite ongoing nalfurafine therapy resulted in rapid and sustained symptom relief with favorable tolerability. The cross-sectional survey also revealed a high prevalence of CKD-associated pruritus in our hemodialysis population, while a substantial proportion of patients receiving nalfurafine continued to experience moderate-to-severe symptoms despite treatment. In the present study, 80.5% of patients reported persistent pruritus, a prevalence that was broadly consistent with previous reports [5,6]. In contrast, 67% of patients experienced moderate-to-severe pruritus despite combination therapy, which was somewhat higher than the approximately 40% reported in previous studies [14]. This discrepancy may reflect differences in patient characteristics, dialysis-related factors, and the extent of non-pharmacological management. One possible explanation is that, before implementation of the treatment algorithm used in the present study, systematic skin care, including the appropriate and consistent use of moisturizers, as well as lifestyle guidance regarding bathing habits, may not have been sufficiently provided to all patients. Optimization of these basic supportive measures may therefore represent an important component of comprehensive pruritus management. In addition, the proportion of patients undergoing online hemodiafiltration at our clinic was 32.4%, which was considerably lower than the nationwide HDF prevalence of nearly 60% (59.1%, with online HDF accounting for 68.3% among HDF modalities) in Japan [15]. Differences in dialysis modality and efficiency may influence the removal and accumulation of uremic toxins and middle molecules, including β2-microglobulin, and may have contributed, at least in part, to the relatively high prevalence of persistent symptoms in our cohort. Taken together, these findings underscore the heterogeneous and multifactorial nature of CKD-associated pruritus. They further suggest that this condition is unlikely to be adequately managed using a uniform, one-size-fits-all pharmacological approach. Rather, treatment should be individualized and optimized according to each patient’s underlying pathophysiological profile, symptom severity, response to previous therapies, dialysis conditions, and susceptibility to adverse events [16]. Such an individualized approach may be particularly important for identifying patients who are unlikely to achieve sufficient symptom control with conventional therapy and who may benefit from switching to an alternative agent with distinct pharmacological characteristics.

The clinical significance of this study lies in its identification of patients with an inadequate response to existing therapy and in its evaluation of a patient-stratified switch to an agent with distinct pharmacological properties under real-world clinical conditions. In the switch group, mean NRS scores decreased significantly from 5.9 ± 1.7 at baseline to 2.3 ± 2.3 at week 4 and 2.0 ± 0.9 at week 16, with improvement observed in every patient. In contrast, the control group showed no significant change over the same observation period, suggesting that the improvement observed after switching to difelikefalin was unlikely to be explained by spontaneous symptom fluctuation or other nonspecific temporal effects. Although the efficacy of difelikefalin has already been established in pivotal phase III trials, the present study provides complementary real-world evidence by focusing specifically on patients with an inadequate response to nalfurafine and by extending follow-up to 16 weeks [17]. This is clinically relevant because treatment response in patients with refractory CKD-associated pruritus may differ from that observed in broader trial populations, and information from routine clinical practice remains relatively limited. The sustained improvement observed over the course of the present study is also consistent with findings from previous clinical trials. In the 12-week international phase III KALM-1 trial, the proportion of patients achieving the primary endpoint, defined as an improvement of at least 3 points, was significantly higher in the difelikefalin group than in the placebo group, with symptom improvement emerging early after treatment initiation and being maintained through week 12 [17]. In addition, Japanese clinical trials and their long-term extension studies have shown that the response rate exceeded 50% by week 4, increased to nearly 70% by week 6, and that approximately 50–70% of patients experienced clinically meaningful improvement with continued treatment for 12–16 weeks [9,14]. These findings suggest that the therapeutic effect of difelikefalin may continue to evolve beyond the first several weeks of treatment and that an early partial response does not necessarily indicate insufficient long-term efficacy. Accordingly, to maximize therapeutic benefit, continuation of difelikefalin for at least 12–16 weeks should be considered even when the initial response at week 4 is modest, provided that treatment remains well tolerated. This approach may allow a more appropriate assessment of mid- to long-term efficacy and may help avoid premature discontinuation in patients who could ultimately derive meaningful symptomatic benefit from continued therapy.

The pathogenesis of CKD-associated pruritus is complex and multifactorial, involving a broad range of mechanisms, including xerosis, the accumulation of uremic toxins, systemic inflammation, disturbances in mineral metabolism, peripheral nerve sensitization, and dysregulation of the endogenous opioid system [16,18]. Consequently, even among patients with comparable NRS scores, the predominant pathophysiological mechanisms underlying pruritus may differ substantially, potentially resulting in considerable interindividual variability in treatment response. Within the endogenous opioid system, an imbalance between itch-inhibitory kappa-opioid receptor signaling and itch-promoting mu-opioid receptor signaling is considered to play an important role in the development and persistence of CKD-associated pruritus [18]. This pathophysiological heterogeneity may partly explain why a therapeutic agent that is effective in one patient may provide insufficient symptom relief in another, despite apparently similar clinical severity. Difelikefalin is a peripherally selective kappa-opioid receptor agonist with minimal penetration of the blood–brain barrier, allowing modulation of peripheral itch signaling while potentially reducing the risk of centrally mediated adverse effects [19]. The favorable response to difelikefalin observed in patients who had responded inadequately to nalfurafine is therefore of particular clinical interest. Although both agents target kappa-opioid receptors, differences in blood–brain barrier permeability, peripheral versus central pharmacological activity, receptor selectivity, pharmacokinetic characteristics, and route of administration may contribute to differences in clinical response. Nevertheless, the observational design and small sample size of the present study do not allow definitive conclusions regarding the mechanisms responsible for the improved response after switching to difelikefalin [9]. Further mechanistic and prospective studies are warranted to determine which patient characteristics are associated with a preferential response to each therapeutic agent. These findings support a precision medicine approach in which treatment selection is guided by matching individual clinical phenotypes, previous treatment responses, and potential adverse-event risks with the specific pharmacological characteristics of each drug, rather than considering agents within the same pharmacological class to be clinically interchangeable. Such an approach may be particularly relevant to CKD-associated pruritus because of its heterogeneous pathophysiology and the considerable variability in treatment responsiveness among patients. Furthermore, difelikefalin is administered intravenously at the end of each hemodialysis session, thereby eliminating dependence on daily oral medication adherence and allowing treatment to be readily incorporated into routine dialysis workflows. This mode of administration may represent a practical advantage in the hemodialysis population, in whom polypharmacy, advanced age, cognitive or functional limitations, and multiple comorbidities may complicate adherence to oral treatment regimens. Reliable administration under the supervision of dialysis staff may therefore improve treatment continuity and reduce the burden associated with additional oral medications. Accordingly, post-dialysis intravenous administration of difelikefalin may offer an adherence-related advantage for long-term treatment maintenance, particularly in patients with complex medication regimens. However, medication adherence and treatment persistence were not directly evaluated in the present study, and this potential advantage should therefore be regarded as a clinically plausible hypothesis rather than a demonstrated benefit [14].

Safety observations from the present study further support the rationale for tailoring antipruritic therapy according to individual patient characteristics and risk profiles. Although oral nalfurafine is an established and effective treatment option for CKD-associated pruritus, its ability to penetrate the central nervous system may result in centrally mediated adverse events, including somnolence and dizziness, which can limit dose escalation or, in some patients, necessitate treatment discontinuation [20]. In contrast, difelikefalin is characterized by peripheral kappa-opioid receptor selectivity and minimal penetration of the blood–brain barrier, pharmacological properties that may reduce the risk of central nervous system-mediated adverse effects [21,22]. In the present cohort, no severe adverse events were considered clearly attributable to difelikefalin, and no central nervous system-related adverse events requiring treatment discontinuation were observed during the study period. These findings are particularly relevant in the hemodialysis population, in whom advanced age, multiple comorbidities, and polypharmacy may increase susceptibility to drug-related adverse events. Although one patient experienced worsening somnolence after switching to difelikefalin, the symptom resolved promptly following discontinuation of newly prescribed pregabalin, making a direct causal relationship with difelikefalin unlikely. Patients undergoing maintenance dialysis frequently receive multiple concomitant medications for neuropathic pain, sleep disturbances, cardiovascular disease, and other comorbid conditions. Centrally acting agents, including gabapentinoids, may increase the risk of somnolence, impaired alertness, and falls, particularly in older patients and those with reduced drug clearance [23]. Consequently, attributing neurological symptoms to a newly introduced antipruritic agent may be challenging in routine clinical practice, and careful assessment of concomitant medications and temporal relationships between drug initiation and symptom onset is essential. When selecting an antipruritic agent, clinicians should therefore comprehensively evaluate not only the severity and clinical burden of pruritus but also the patient’s age, comorbidities, concomitant medications, hemodynamic stability, previous treatment response, and history of adverse events. Particular attention should also be given to cardiovascular and hemodynamic status. In the patient who discontinued difelikefalin because of intradialytic hypotension, subsequent investigations revealed significant underlying coronary artery disease and valvular heart disease, and the hypotensive episodes resolved following appropriate treatment of these cardiovascular conditions. The clinical course therefore suggested that difelikefalin was unlikely to have been the primary cause of the intradialytic hypotension. Nevertheless, hemodynamic fluctuations have been reported in clinical trial data, and a potential contribution of difelikefalin cannot be completely excluded based on this single observation [24]. Careful monitoring during treatment initiation is therefore warranted, particularly in patients with pre-existing hemodynamic instability or substantial cardiovascular comorbidity. Taken together, these safety observations emphasize the importance of interpreting adverse events within the broader clinical context rather than attributing them solely to the newly introduced therapy. Such comprehensive risk assessment and patient stratification are consistent with the principles of individualized medicine and may help optimize the balance between therapeutic efficacy and safety in the management of CKD-associated pruritus.

This study has several limitations. First, it was a single-center retrospective observational study with small sample sizes in both the switch and control groups (n = 8 each), limiting statistical power and generalizability. Furthermore, the baseline NRS score was higher in the switch group than in the control group (5.9 ± 1.7 vs. 4.6 ± 1.3). Given the small sample size, regression-to-the-mean cannot be entirely excluded as a contributing factor to the observed reduction in NRS scores in the switch group. Larger multicenter studies are required to confirm these findings. Second, an ideal comparator would have been patients who continued nalfurafine without switching to difelikefalin, because such patients would have shared prior nalfurafine exposure and a more similar clinical context with the switch group. However, among the 14 patients receiving nalfurafine, only four were not switched during the observation period: one patient had mild pruritus with adequate symptom control under nalfurafine therapy, whereas the remaining three had moderate-to-severe pruritus based on NRS assessment but reported no impairment in daily activities and did not change treatment after consultation with their attending physicians. These patients therefore did not represent an appropriate comparator population for evaluating the effect of switching in clinically burdensome, refractory pruritus. Accordingly, to account for the potential influence of seasonal variation on pruritus, eight patients receiving neither nalfurafine nor difelikefalin were matched and selected as controls. Although matching was performed for major baseline characteristics, including age, sex, dialysis vintage, baseline NRS score, and the same observation period, residual confounding may have remained because of differences in treatment indications, prior treatment history, and clinical decision-making. Third, the primary endpoint, the NRS score, was a patient-reported outcome and may have been affected by reporting bias or placebo effects associated with switching from an oral medication to post-dialysis intravenous administration. Fourth, changes in concomitant medications or clinical conditions during the observation period may have influenced symptom and safety evaluations, as exemplified by transient pregabalin use in the present study. Fifth, secondary parameters such as sleep quality, objective dermatological scales, patient satisfaction, overall quality-of-life metrics, and biomarkers were not systematically evaluated. Future studies should identify biomarkers and clinical predictors of difelikefalin responsiveness. Predictive models integrating patient background, dialysis conditions, laboratory data, and prior treatment history may help refine treatment algorithms and transition CKD-associated pruritus management from empirical stepwise therapy to precision-based therapeutic selection.

## 5. Conclusions

In this single-center retrospective study, switching from nalfurafine to difelikefalin was associated with rapid and sustained improvement in refractory pruritus among patients receiving maintenance hemodialysis, without severe adverse events considered directly attributable to difelikefalin. These findings suggest that difelikefalin may be a useful therapeutic option for patients with an inadequate response to nalfurafine, particularly when treatment selection is individualized according to prior treatment response, symptom severity, comorbidities, adverse-event risk, and route of administration. Given the small sample size, retrospective design, and limitations related to control-group selection, larger prospective multicenter studies incorporating clinical predictors and patient-reported outcomes are warranted to validate these findings and establish optimized treatment algorithms for refractory hemodialysis-associated pruritus.

## Figures and Tables

**Figure 1:**
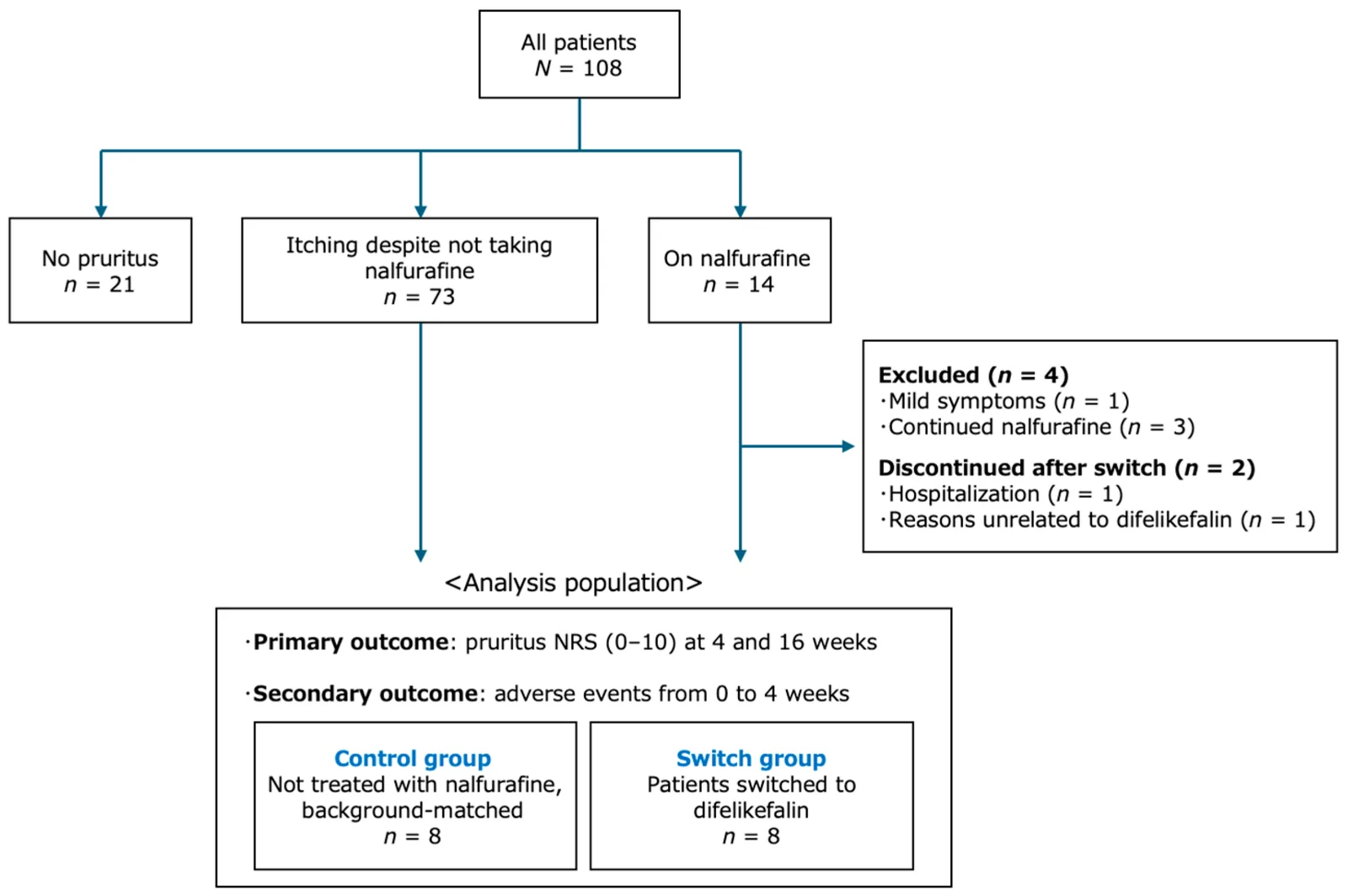
**Patient selection and study-group allocation.** All 108 patients receiving maintenance hemodialysis were assessed for pruritus severity using the NRS. Of the 14 patients receiving nalfurafine, 10 were switched to difelikefalin because of persistent moderate-to-severe pruritus, of whom eight completed the efficacy and safety assessments. Eight patients receiving neither nalfurafine nor difelikefalin during the observation period were selected as matched controls. NRS, numerical rating scale.

**Figure 2:**
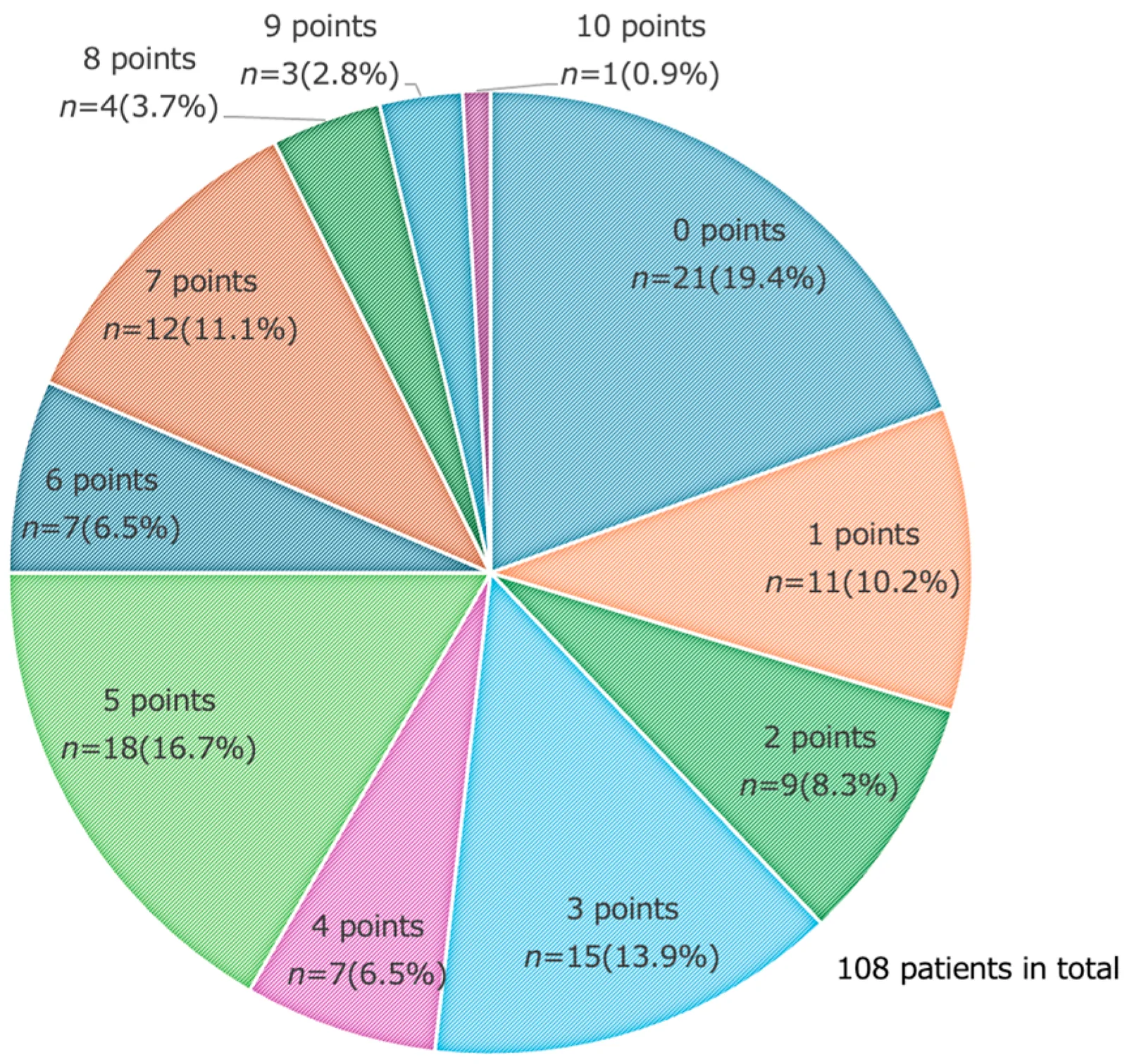
**Distribution of NRS Scores Among All Patients.** The pie chart shows the number and percentage of patients at each NRS score from 0 to 10 (*n* = 108). NRS, numerical rating scale.

**Figure 3:**
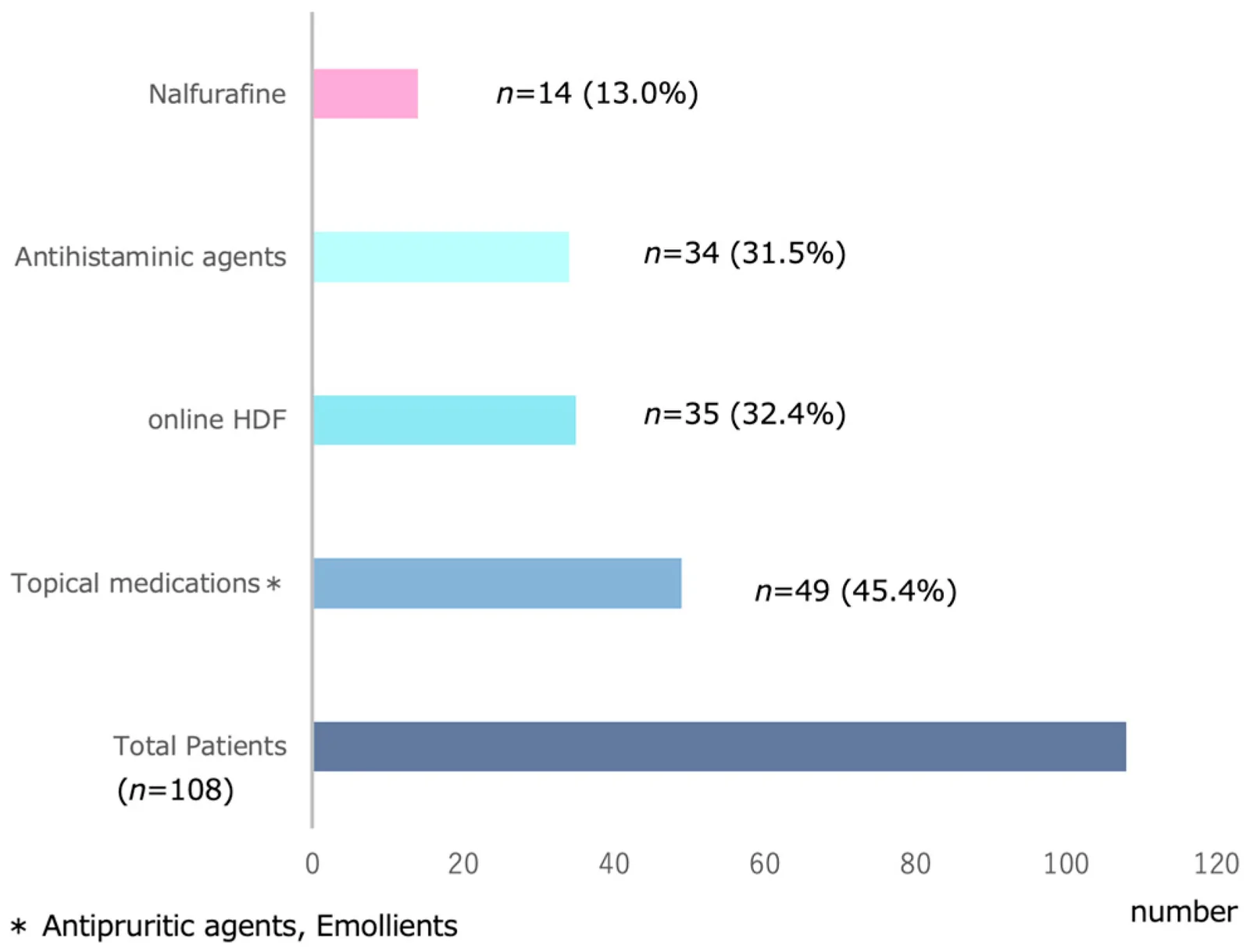
**Details of treatment regimens for pruritus.** The bar chart shows the numbers and proportions of patients receiving topical medications, online hemodiafiltration (HDF), antihistamines, and nalfurafine among all eligible patients (*n* = 108). * Topical medications included antipruritic agents and emollients.

**Figure 4:**
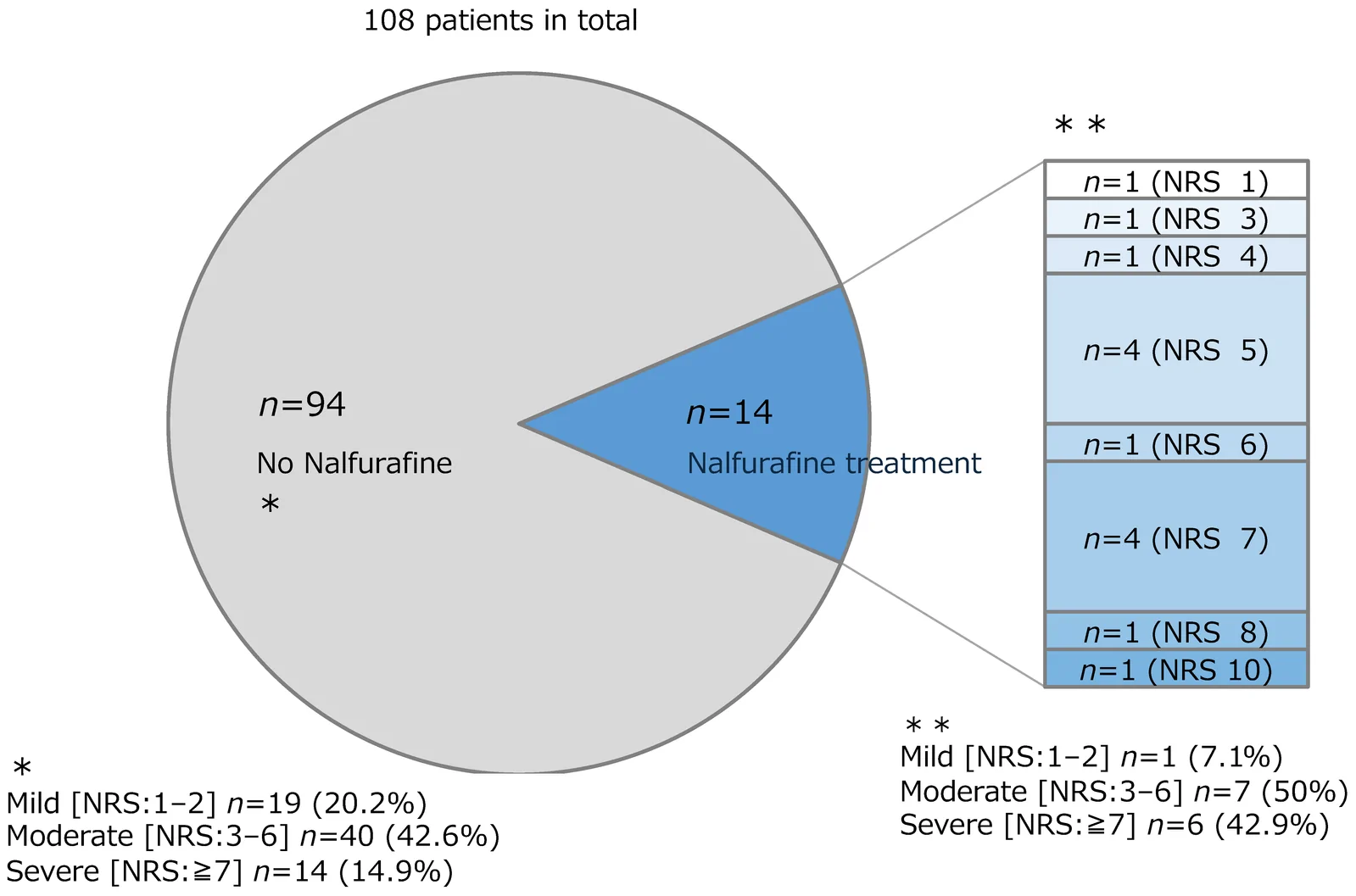
**Nalfurafine Treatment Rates and NRS Score Distribution**. Of the 108 eligible patients, 14 received nalfurafine and 94 did not. The secondary pie chart shows the distribution of NRS scores among patients receiving nalfurafine. NRS, numerical rating scale.

**Table 1:** Demographic, Clinical, and Biologic Characteristics of the Patients According to Treatment Group.

Characteristics (Mean ± SD)	Total (*n* = 16)	Switch Group (*n* = 8)	Control (*n* = 8)	*p* Value (Switch vs. Control)
Age	72 ± 14.9	72 ± 12.2	73 ± 10.2	0.8106
Female Sex (%)	3(18.8%)	1(12.5%)	2(25.0%)	0.4103
Dry Weight	57.4 ± 14.9	54.3 ± 9.8	60.5 ± 19.0	0.4273
Duration of Dialysis	9.6 ± 6.9	8.0 ± 6.3	11.3 ± 7.5	0.3625
itchiness (NRS) Pre-treatment	5.3 ± 1.6	5.9 ± 1.7	4.6 ± 1.3	0.1244
Hb (g/dL)	10.7 ± 0.7	10.6 ± 0.7	10.9 ± 0.6	0.4000
Alb (g/dL)	3.6 ± 0.3	3.4 ± 0.3	3.7 ± 0.3	0.1585
BUN (mg/dL)	49.5 ± 8.4	51.0 ± 9.1	48.0 ± 8.0	0.4961
Cr (mg/dL)	9.1 ± 2.1	9.2 ± 1.8	9.0 ± 2.5	0.8451
Corrected Ca (mg/dL)	9.3 ± 0.4	9.3 ± 0.5	9.3 ± 0.3	0.4486
P (mg/dL)	5.0 ± 1.0	5.1 ± 0.9	4.9 ± 1.1	0.6839
intact PTH (pg/dL)	111 ± 93.0	105 ± 64.0	116 ± 119.9	0.8103

NRS; numerical rating scale, Hb; hemoglobin, Alb; albumin, BUN; blood urea nitrogen, Cr; creatinine, Ca; calcium, P: phosphorus, PTH; parathyroid hormone.

## Data Availability

The raw data supporting the findings of this study are not publicly available and will not be provided because they contain information that could compromise patient privacy. Data containing information that could identify individual patients will not be shared.
